# Internet chemotherapy information is of good quality: assessment with the DISCERN tool

**DOI:** 10.1038/bjc.2012.223

**Published:** 2012-06-07

**Authors:** R Som, N P Gunawardana

**Affiliations:** 1 Department of General Surgery, Wycombe Hospital, Queen Alexandra Road, Wycombe HP11 2TT, UK; 2 National Hospital for Neurology and Neurosurgery, Queen Square, London WC1N 3BG, UK

**Sir,**

We read with interest Davies and Yeoh’s (2012) paper about internet chemotherapy information (ICI). The pertinent points the authors make are the internet’s potential as a source of information for patients, but also the concerns of health professionals (HP) that ICI is inaccurate and can cause harm to patients who use it.

We performed a search of the term ‘chemotherapy’ on google.com, and then sought to assess the quality of the first 10 websites for this search using the DISCERN instrument. Google.com was selected as the search engine of choice because it is the most commonly used search engine (Burns, 2012). Sponsored links and news articles were excluded. The DISCERN instrument is a validated rating tool of the quality of health information (Charnock *et al*, 1999). It is available for use by both HPs and the general public. Websites are given a score out of 80 based on 15 questions regarding the sites’ content. The questions are rated on a 5-point scale from ‘no’ (1 point) to ‘yes’ (5 points). The 10 websites were independently evaluated with the DISCERN instrument by two of the authors of this letter (RS and NG). Discrepancies between the scores were discussed with a view to reaching a consensus.

The mean DISCERN score was 56.1 (s.d.=8.76). The DISCERN handbook would therefore categorise these websites as being of ‘good quality’ (excellent=63–75; good=51–62; fair=39–50; poor=27–38; very poor=15–26). The range of scores was 41–69, so all of the top 10 sites are of ‘fair quality’ at least. Four of these sites are ‘excellent’. The websites scored highest for question 15 of the criteria – ‘Does it (the publication/website) provide support for shared decision making?’ – with a mean score of 4.7, and question 13 – ‘Does it describe how the treatment choices affect quality of life?’, with a mean score of 4.5.

The websites scored lowest for questions 5, 8 and 12:

- ‘Is it clear when the information used or reported in the publication was produced?’
- ‘Does it refer to areas of uncertainty?’
- ‘Does it describe what would happen if no treatment is used?’

The scores for these criteria are 2.5, 2.7 and 2.7, respectively.

Our evaluation shows that the overall quality of ICI is good. However, Davies and Yeoh (2012) found that the majority of HPs do not routinely recommend ICI to their patients, and fear that it could cause harm to them, partly because HPs perceive it to be of poor quality. This suggests that HPs’ mistrust of the ICI is misplaced, and that the patients might benefit from HPs openly recommending ICI.

However, the concerns of HPs are not completely unfounded. There are several tools and methods for evaluating website quality, such as the HONcode principles (Boyer *et al*, 1998) and the JAMA benchmarks (Silberg *et al*, 1997), as well as the DISCERN criteria. Further systematic evaluations are warranted to explore the quality of ICI.
